# Interaction between *TP53* and *XRCC1* increases susceptibility to cervical cancer development: a case control study

**DOI:** 10.1186/s12885-018-5149-0

**Published:** 2019-01-07

**Authors:** Gui-Cen Liu, Yun-Fei Zhou, Xiao-Chao Su, Jun Zhang

**Affiliations:** Institute of Molecular Medicine and Oncology, Chongqing Medical University, Yuzhong District, Chongqing City, 400016 China

**Keywords:** Cervical cancer, *XRCC1*, *TP53*, *FGFR3*, SNP-SNP interaction

## Abstract

**Background:**

Cervical cancer is the 4th highest cause of female reproductive tract malignancies. Multiple loci have been identified as important determinant factors for tumor susceptibility. In this report, we aimed to explore the roles of gene polymorphisms affecting x-ray repair cross complementing 1 (XRCC1), the tumor protein p53 (TP53), and fibroblast growth factor receptor 3 (FGFR3) in the context of susceptibility to cervical cancer. Additionally, we assessed the impact of single nucleotide polymorphism-single nucleotide polymorphism (SNP-SNP) interaction of these three genes in the context of cervical cancer risk in Chinese women.

**Methods:**

A case-control study consisted of 340 women located in Chongqing. Of these women, 121 were diagnosed with cervical cancer, 118 served as healthy controls, and 101 were specifically recruited elderly patients above the age of 80 who showed no history of cervical cancer. Three SNPs (*XRCC1* rs25487, *TP53* rs1042522, and *FGFR3* rs121913483) were examined using mutation analysis of mismatch amplification PCR (MAMA-PCR) on samples obtained from peripheral blood.

**Results:**

Our results indicated that females from southwestern China all exhibited a wild-type phenotype at *FGFR3* rs121913483. We also observed that the rs25487 mutation was significantly increased within the cervical cancer population. A 2-locus SNP-SNP interaction pattern (rs25487 and rs1042522) was significantly associated with cervical cancer risk (cases vs. negative controls: OR = 4.63, 95% CI = 1.83–11.75; cases vs. elderly group: OR = 17.61, 95% CI = 4.34–71.50).

**Conclusions:**

This is the first study to identify a novel interaction between the *XRCC1* and *TP53* genes that is highly associated with susceptibility to cervical cancer risk in a female population in southwestern China.

**Electronic supplementary material:**

The online version of this article (10.1186/s12885-018-5149-0) contains supplementary material, which is available to authorized users.

## Background

Cervical cancer is the fourth most common malignant tumor of the female reproductive tract, with an estimated 528,000 new cases and 266,000 deaths occurring annually worldwide [1]. Among the less-developed nations, this disease accounts for 85% of the prevalence and 87% of the mortality [2]. Infection by Human papillomavirus (HPV) is the primary cause of the vast majority of cervical tumors [3, 4]; however, most HPV infections are temporary, and more than 90% of infections are cleared by hosts within 3 years. Additionally, only a small portion of HPV-infected women develop cervical cancer [5, 6]. A number of studies have shown that HPV infection alone is insufficient to cause tumorigenesis and that tumor development is triggered by the combined effects of environmental stimuli and individual genetic factors [7, 8]. SNPs are considered to be the most widespread type of genetic variation (approximately 90%) in the human genome [9]. Examining SNP loci in the context of patients is essential for developing effective personalized medicine [10].

Recently, numerous studies have been performed to assess the effect of SNPs on cervical cancer susceptibility [11–13]. Based on these reports and findings from bioinformatics studies exploring high risk factors within the Chinese population for cervical cancer, we focused our current study on crucial genes within the DNA repair and recombination pathways. Base excision repair (BER) is an important pathway that has been suggested to be crucial for the prevention of cervical cancer [14]; however, the effects of SNP-SNP interaction between genetic polymorphisms within the BER pathway on cervical cancer susceptibility remain unknown. XRCC1 is an important regulator of the BER pathway and TP53 also participates in DNA repair progression. The exact role of TP53 in the formation of cervical cancer, however, remains largely controversial [15–17]. No studies have previously reported a correlation between *FGFR3* polymorphisms within the Chinese population and a genetic predisposition to cervical cancer. Also, data describing the relationships between *FGFR3* polymorphisms and a genetic predisposition to cervical cancer have not been reported in China [18, 19]. Here, the genes encoding XRCC1, TP53, and FGFR3 were chosen for our analysis.

In our current study, we aimed to integrate bioinformatics methods with samples exhibiting diverse clinical features in an effort to explore the roles of various SNPs in the pathogenesis of cervical cancer within the southwestern population of China.

## Methods

### Subjects

Biological specimens were collected from women of similar ethnic and geographical backgrounds in Chongqing, China. A total of 121 women (age: 42.01 ± 9.4) with a history of cervical cancer were recruited from the first affiliated hospital of Chongqing Medical University. The control group consisted of 118 healthy women (age: 35.43 ± 10.41) who were selected following a regular gynecological examination. Another control group was comprised of 101 healthy elderly women with a lifelong proven absence of cervical cancer (aged: 84.5 ± 2.37) (Additional file 1). The number of HPV-positive women was 140 (mean ± SD, 60.68 ± 1.81). No more than 8 (6%) patients had prior screening and 11(8%) patients in the HPV-positive group reported a history of sexually transmitted disease (STD). The number of HPV-negative controls was 197 (mean ± SD, 41.58 ± 0.94), and 8 of these reported a previous history of other undetermined cervical infections (Additional file 2). Samples of 2 ml peripheral blood were obtained from all subjects. The local ethics committee of the Chongqing Medical University approved the experimental design, clinical data collection methods, and data evaluation strategies. These procedures were carried out in accordance with the approved guidelines. Written informed consent was obtained from all participants.

### Gene selection

**Fig. 1:**
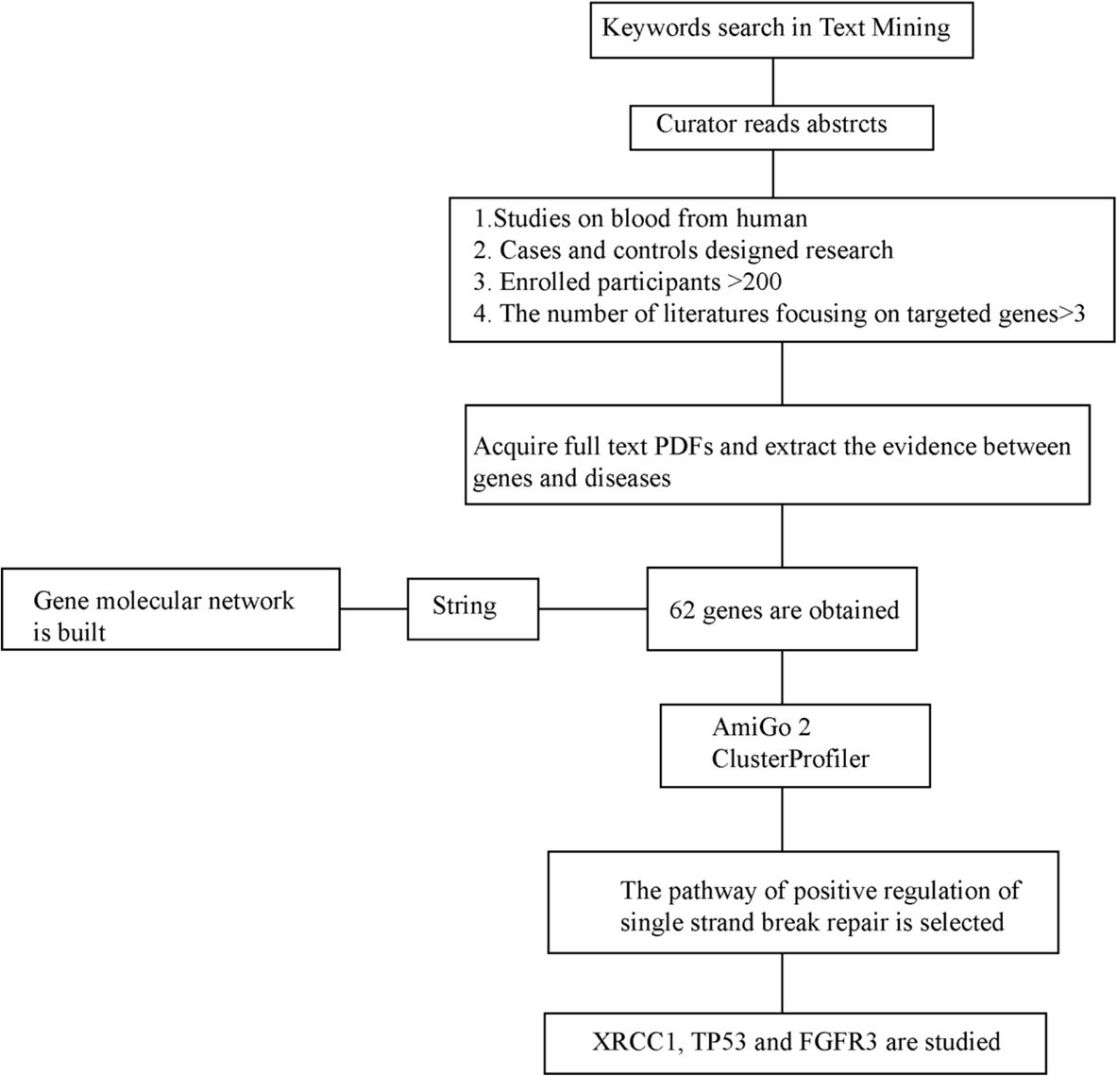
schematic illustration for text mining of dominant SNPs related to pathogenesis of cervical cancer, and identified *XRCC1*, *TP53,* as well as *FGFR3*

All published literature through 31 October 2015 detailing susceptibility to cervical cancer was retrieved by text mining and obtained from the NCBI database. The keywords ‘cervical cancer’ and ‘gene’, ‘single nucleotide polymorphism’, ‘DNA polymorphism’, ‘genetic polymorphism’, or ‘genetic variation’ were used in combination to retrieve the relevant literature. Genes reported by three or more articles with enrolled sample numbers greater than 200 were considered. We e-mailed authors to obtain full text articles if these could not be downloaded from PubMed and Web of Science. According to the literature, there were 62 gene polymorphisms associated with cervical cancer risk. A protein molecular network based on these 62 genes was built by String [20]. AmiGo 2 [21] and ClusterProfiler packages were used to determine enriched GO terms (Fig. 1) [22].

### Isolation of genomic DNA

MAMA-PCR was performed for genotype identification. Genomic DNA was extracted from peripheral blood samples using a Rapid Blood Genomic DNA Isolation Kit (Sangon Biotech, Shanghai, China), and extracts were stored at − 20 °C until use. DNA quantity was measured using an ultraviolet spectrophotometer at 260 nm, and the DNA quality was assessed by agarose gel electrophoresis. The rs121913483 (C > G), rs25487 (G > A), and rs1042522 (G > C) genotypes were identified separately by MAMA-PCR and then validated by Sanger sequencing (Sangon Biotech, Shanghai, China) as described in Additional file 3: Figure S1. The PCR primers (Invitrogen, Shanghai, China) used to amplify the genes are as follows:

The final reaction mixture contained 50 ng template DNA, 0.2 μL Taq polymerase, 2.4 μL dNTPs, 2 μL 10X PCR Buffer (Takara, Japan), 50 nmol each reverse and forward primers, MgCl_2_ (Takara, Japan; FGFR3, 1 μL; XRCC1, 1.5 μL; TP53, 2 μL), and double-distilled H_2_O at a final reaction volume of 20 μL. Amplification conditions for *XRCC1* consisted of an initial denaturing step at 94 °C followed by 36 cycles at 94 °C for 10 s, 57 °C for 30, s and 72 °C for 1 min. MAMA-PCR of *TP53* was performed for 35 cycles at 98 °C for 10 s, 57 °C for 30 s, and 72 °C for 1 min. PCR conditions for *FGFR3* included initial denaturation at 94 °C for 5 min followed by 35 cycles at 94 °C for 30 s, 56 °C for 30 s, and 72 °C for 1 min. All PCR reactions were followed by a final extension at 72 °C for 10 min.

**Table:** 

**Genes**	**Items**	**nucleotide sequence**
**FGFR3**	**F1**	**5’-CCCCCACAGAGCGCTC-3’**
	**F2**	**5’-CCCCCACAGAGCGCTG-3’**
	**R**	**5’-TAGACCCAAATCCTCACGCA-3’**
**TP53**	**F1**	**5’-CAGAGGCTGCTCCCCG-3’**
	**F2**	**5’-CAGAGGCTGCTCCCCC-3’**
	**R**	**5’-AGCCAAGGAATACACGTGGA-3’**
**XRCC1**	**F**	**5’-CTCTGTCTGTCTCCCCTGTCT-3’**
	**R1**	**5’-CGTGTGAGGCCTTACCTCC-3’**
	**R2**	**5’-CGTGTGAGGCCTTACCTCT-3’**

### Statistical analysis

The mean and standard deviation (SD) was used for describing normally distributed continuous variables. Deviations from the Hardy-Weinberg equilibrium (HWE) for three SNPs were tested using a chi-square (χ^2^) test. The association among *TP53*, *XRCC1*, and *FGFR3* and the effects of these associations in the context of cervical cancer risk was studied by calculating the ORs and 95% CIs based on χ^2^ analysis. The SNP-SNP interaction experiments were performed by generalized multifactor dimensionality reduction (GMDR) [23] and logistic regression using SPSS 19.0 software (SPSS Inc., Chicago, IL, USA). A *p*-value of < 0.05 was considered to indicate significance.

## Results

### Identification of candidate SNPs using text mining and bioinformatics

**Fig. 2:**
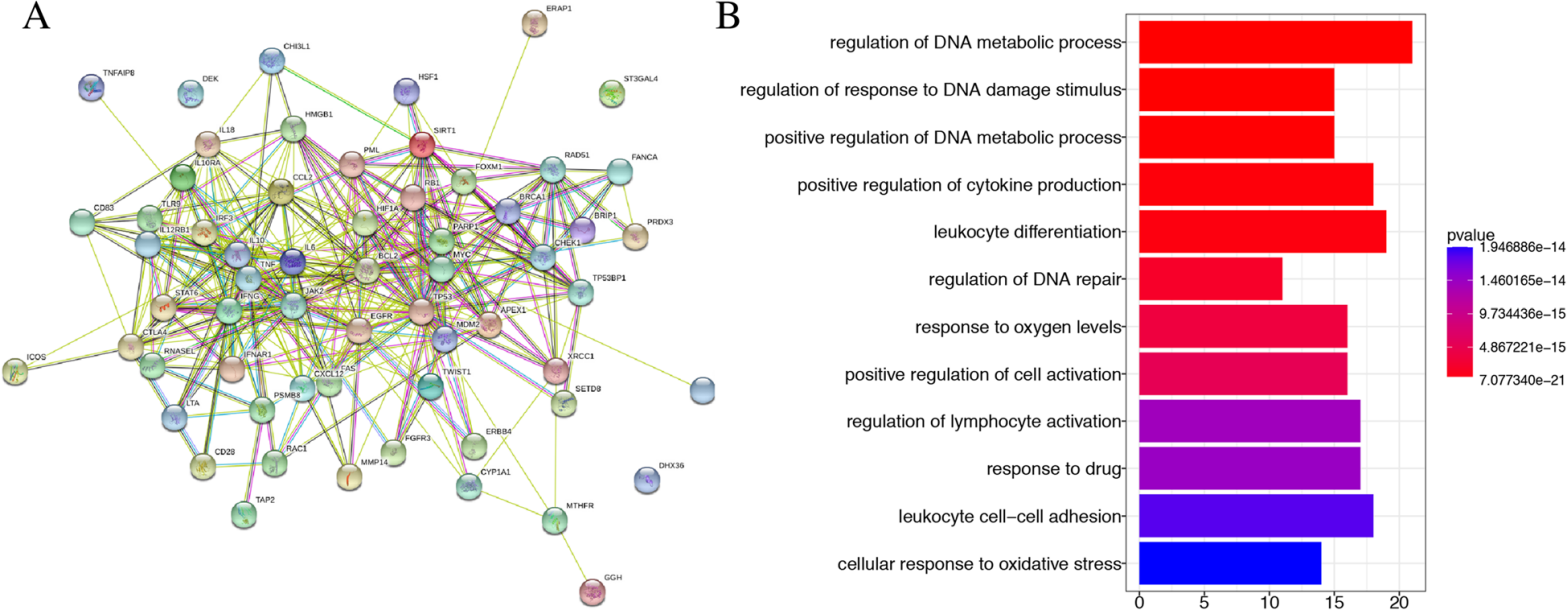
Network analysis of essential protein from String (**a**), and pathway analysis for these 62 genes by ClusterProfiler (**b**)

A molecular network diagram of 62 selected genes related to increased risk of cervical cancer was constructed using String (Fig. 2a). The top three GO items showing the lowest *p*-value included regulation of DNA metabolic processes, regulation of response to DNA damage stimulus, and positive regulation of DNA metabolic processes promoting DNA single strand break repair (Fig. 2b). Here, we focused mainly on this repair pathway and selected genes important for pathway function. It has been confirmed that the XRCC1 gene plays an important role in modulating the risk for cervical cancer [24, 25]; however, the impact of the TP53 gene on this risk is less clear. No results have yet been reported concerning the interaction between XRCC1 and TP53 in the context of cervical cancer. Additionally, the FGFR3 gene, which has no known role in cervical cancer, was chosen for subsequent analysis. In summary, based on bioinformatics studies were selected the XRCC1, TP53, and FGFR3 genes as candidates for this study.

### Genetic analysis

We sequenced the three genes containing rs121913483 (C > G), rs1042522 (G > C), and rs25487 (G > A) from 121 Chinese women suffering from cervical cancer and from 219 controls. The distribution frequencies of rs1042522 and rs25487 were found to be in HWE (Table 1). No variation in rs121913483 was found in any of the cancer cases, negative controls, or the elderly group.

**Table 1:** The genotype distribution and H-W of XRCC1 (rs25487), TP53 (rs1042522) and FGFR3 (rs121913483)

Variable	Alleles	Phenotype	N	HWE	MAF	M/M (%)	M/m (%)	m/m (%)	M/M +M/m	M/m +m/m	Allele M	Allele m
rs25487	G > A	Cases	121	0.18	0.40	47(38.9)	51(42.1)	23(19)	98	74	145(59.9)	97(40.1)
		NC	118	0.15	0.25	70(59.3)	38(32.2)	10(8.5)	108	48	178(75.4)	58(24.6)
		OC	101	0.45	0.27	55(54.4)	37(36.6)	9(9)	92	46	147(72.8)	55(27.2)
rs1042522	G > C	Cases	121	0.18	0.38	50(41.3)	50(41.3)	21(17.4)	100	71	150(62)	92(38)
		NC	108	0.74	0.42	37(34.3)	51(47.2)	20(18.5)	88	71	125(57.9)	91(42.1)
		OC	99^a^	0.07	0.44	35(35.4)	40(40.4)	24(24.2)	75	64	110(55.6)	88(44.4)
rs121913483	G > C	Cases	117			117	0	0				
		NC	113			113	0	0				
		OC	89			89	0	0				

*N* Number, *HWE* Hardy-Weinberg equilibrium, *MAF* Minor allele frequency, *NC* Negative controls, *OC* Old controls

^a^The number of subjects in each group at different sites may be different due to insufficient DNA

A case-control study of the three SNPs was conducted to compare the cervical cancer group to the elderly control group. Table 2 illustrates the distribution of *XRCC1* rs25487 and *TP53* rs1042522 within the study groups. Here, we found that the minor alleles of the XRCC1 gene significantly correlated to the cervical cancer group when compared to the negative group (OR = 3.43, 95% CI = 1.50–7.85, *p* = 0.00) or the elderly group (OR = 3.00, 95% CI = 1.26–7.10, *p* = 0.03). This genotype (dominant model) conveyed a nearly 2-fold higher risk compared with that of negative controls. No significant differences were observed among our experimental groups in regard to TP53 gene status.

**Table 2:** The association between gene polymorphism of XRCC1 (rs25487), TP53(rs1042522) and cervical cancer

Variable	Groups	Co-dominate model				Recessive model		
			*P*-value	OR	95%CI	*P*-value	OR	95%CI
rs25487	Cases vs NC	G/A	0.00^*^	2.00	1.14–3.50	0.02^*^	2.54	1.15–5.59
		A/A		3.43	1.50–7.85			
	Cases vs OC	G/A	0.03^*^	1.61	0.91–2.87	0.03^*^	2.40	1.06–5.46
		A/A		3.00	1.26–7.10			
	NC vs OC	G/A	0.76	0.81	0.45–1.43	0.91	0.95	0.37–2.43
		A/A		0.87	0.33–2.30			
rs1042522	Cases vs NC	G/C	0.54	0.73	0.41–1.29	0.82	0.92	0.47–1.82
		C/C		0.78	0.37–1.64			
	Cases vs OC	G/C	0.41	0.88	0.48–1.59	0.21	0.66	0.34–1.27
		C/C		0.61	0.30–1.27			
	NC vs OC	G/C	0.51	1.21	0.65–2.24	0.32	0.71	0.36–1.39
		C/C		0.79	0.37–1.67			
Variable	Groups	Dominant model			Allele model			
		*P*-value	OR	95%CI	*P*-value	OR	95%CI	
rs25487	Cases vs NC	0.00^*^	2.30	1.37–3.86	0.00^*^	2.05	1.39–3.04	
	Cases vs OC	0.02^*^	1.88	1.10–3.22	0.00^*^	1.79	1.20–2.67	
	NC vs OC	0.47	0.82	0.48–1.40	0.53	0.87	0.57–1.34	
rs1042522	Cases vs NC	0.27	0.74	0.43–1.27	0.37	0.84	0.58–1.23	
	Cases vs OC	0.37	0.78	0.45–1.34	0.17	0.77	0.52–1.12	
	NC vs OC	0.87	1.05	0.59–1.86	0.64	0.91	0.62–1.34	

*OR* Odd ratio, *CI* Confidence interval, *NC* Negative controls, *OC* Elderly patients above the age of 80

*indicated *p*-value< 0.05

### SNP-SNP interaction between *XRCC1* and *TP53* polymorphisms

To assess SNP-SNP interaction, genotypic data for the three polymorphisms detailed in this study were analyzed by GMDR and logistic regression. The SNP-SNP interaction data are summarized in Tables 3 and 4, and the genetic models are presented in Table 5. When the rs25487 and rs1042522 status of both control groups was combined into a single analysis, logistic regression showed a clear relationship between heterogeneity in the ORs among heterozygotes (*p* < 0.05). Examining rs25487 and rs1042522 provided the best two-factor model (testing accuracy (TA) = 0.63, *p* = 0.00, cross-validation consistency (CVC) =10/10), and this model was as effective as the three-factor model that included rs121913483. Given this, we determined that the *XRCC1* rs25487 heterozygote genotype combined with the *TP53* rs1042522 heterozygote genotype was associated with an almost 4-fold (cases vs. negative controls) or 17-fold (cases vs. elderly group) increase in cervical cancer risk (cases vs. negative controls: OR = 4.63, 95% CI = 1.83–11.75, *p* = 0.00; cases vs. elderly group: OR = 17.61, 95% CI = 4.34–71.50, *p* = 0.00).

**Table 3:** The gene - gene interaction of XRCC1 and TP53 in the cervical cancer group and the negative control group

	B	SE	Wald	N	P	OR	95% CI	
Recessive model								
XRCC1	−0.15	0.88	0.03	1	0.86	0.86	0.15	4.79
Tp53	0.29	0.89	0.11	1	0.75	1.33	0.24	7.56
Tp53-GG/GC by XRCC1-GG/GA	−0.06	0.96	0.00	1	0.95	0.95	0.15	6.16
常数	0	0.82	0	1	1	1		
Additive model								
Tp53			7.71	2	0.02*			
Tp53-GC	−1.10	0.40	7.69	1	0.01*	0.33	0.15	0.72
Tp53-CC	−0.41	0.51	0.64	1	0.42	0.67	0.25	1.80
Tp53 * XRCC1			10.49	4	0.03*			
Tp53-GC by XRCC1-GA	1.53	0.48	10.43	1	0.00*	4.63	1.83	11.75
Tp53-GC by XRCC1-AA	0.80	0.58	1.93	1	0.17	2.23	0.72	6.92
Tp53-CC by XRCC1-GA	−0.10	0.64	0.02	1	0.88	0.91	0.26	3.20
Tp53-CC by XRCC1-AA	0.11	0.94	0.01	1	0.91	1.11	0.18	6.97
常数	0.30	0.22	1.93	1	0.17	1.35		
Dominant model								
XRCC1-GA/AA	0.71	0.27	6.84	1	0.01*	2.03	1.20	3.46
Tp53-GC/CC	−0.46	0.28	2.69	1	0.10	0.63	0.37	1.09
常数	−0.02	0.25	0.01	1	0.93	0.98		

*indicated *p*-value< 0.05

**Table 4:** The gene - gene interaction of XRCC1 and TP53 in the cervical cancer group and the elderly patients above the age of 80 group

	B	SE	Wald	N	P	OR	95% CI	
Recessive model								
Tp53-GG/GC	1.34	0.55	5.85	1	0.02*	3.81	1.29	11.26
Tp53-GG/GC by XRCC1-GG/GA	−1.07	0.49	4.68	1	0.03*	0.34	0.13	0.90
常数	−0.13	0.30	0.20	1	0.66	0.88		
Additive model								
XRCC1			5.58	2	0.06			
XRCC1-GA	−0.85	0.49	3.03	1	0.08	0.43	0.16	1.11
XRCC1-AA	0.69	0.74	0.87	1	0.35	1.99	0.47	8.42
Tp53			8.74	2	0.01*			
TP53-GC	−1.42	0.48	8.71	1	0.00*	0.24	0.10	0.62
TP53-CC	−0.81	0.57	2.07	1	0.15	0.44	0.15	1.34
Tp53 * XRCC1			17.22	4	0.00*			
TP53-GC by XRCC1-GA	2.87	0.72	16.09	1	0.00*	17.61	4.34	71.50
TP53-GC by XRCC1-AA	1.22	1.05	1.34	1	0.25	3.37	0.43	26.29
TP53-CC by XRCC1-GA	0.95	0.81	1.37	1	0.24	2.57	0.53	12.51
TP53-CC by XRCC1-AA	−0.49	1.19	0.17	1	0.68	0.62	0.06	6.32
常数	0.61	0.34	3.17	1	0.08	1.85		
Dominant model								
TP53-GC/CC	−0.96	0.35	7.66	1	0.01*	0.39	0.20	0.76
TP53-GC/CC by XRCC1-GA/AA	1.29	0.37	12.54	1	0.00*	3.64	1.78	7.43
常数	0.36	0.22	2.62	1	0.11	1.43		

*indicated *p*-value< 0.05

**Table 5:** The best model predicted ovarian cancer risk by GMDR

Model	Training bal. acc	Testing bal. acc	Sign test *p* value	CV consistency
XRCC1	0.58	0.58	7(0.17)	10/10
XRCC1 TP53	0.66	0.63	10(0.00*)	10/10
XRCC1 TP53 FGFR3	0.66	0.63	10(0.00*)	10/10

Data was analyzed by GMDR 0.9 with default settings

*indicated *p*-value< 0.05

## Discussion

Three SNPs were selected using text mining and bioinformatics, and they included *FGFR3* rs121913483, *TP53* rs1042522*,* and *XRCC1* rs25487. In our current study, we found that no “C” allele of *FGFR3* rs121913483 exists in any of our experimental groups. The genotype frequency of the *TP53* rs1042522 polymorphism exhibited no obvious relationship to cervical cancer risk based on the frequency distribution of genotypes as indicated by the results from our logistic regression analysis. The “A” allele of rs25487 was considered as a leading allele for an increased risk of cervical cancer, as there is a significant association of *XRCC1* gene polymorphism with cervical cancer. Additionally, the risk for cervical cancer was particularly high when *TP53* and *XRCC1* heterozygotes existed simultaneously. The risk of cervical cancer was 17-fold compared with that of our elderly group.

The *FGFR3* gene, a member of the tyrosine protein kinase family, is located on chromosome 4p16.3. This gene is primarily involved in bone development and osteogenesis [26]. Mutations of *FGFR3* were reported in 3 out of 12 primary French cervical carcinomas [18]. In contrast, these mutations were not found in samples taken from Norwegian patients [19]. The genetic contribution of *FGFR3* to cervical cancer risk was further investigated in the Chinese population. We found no mutation of *FGFR3* in any groups used for our study. It is likely that these discrepancies are a result of racial differences and genetic background diversity.

*XRCC1* is a 33 kb gene located at chromosome 19q13.2–13.3. The encoded protein acts as a scaffolding protein for a number of repair enzymes and functions in enzymatic follow-up steps during DNA repair [27, 28]. The XRCC1 protein has been reported to be involved in single-strand break repair, base excision repair, and nucleotide excision repair [29]. A polymorphic marker (rs25487) is located in exon 10 of this gene and has been examined for possible association with several malignancies, including cervical cancer [30, 31]. Our findings confirm the association of *XRCC1* rs25487 with an elevated risk for cervical cancer.

The *TP53* gene, located on chromosome 17p13.1, encodes a known tumor suppressor [32]. This gene product prevents genomic mutations and functions to conserve gene stability [33]. The relationship between *TP53* and susceptibility to cervical cancer has been contradictory in previous studies. A study by Storey suggested that rs1042522 significantly enhanced the risk for cervical cancer (OR = 7.4, 95% CI = 2.1–29.4) [15], which was consistent with results from a study by Klug (OR = 2.2, 95% CI = 0.6–7.6) [34]. Contrary to these observations, the results of a study by Rosenthal did not find positive correlation between rs1042522 and cervical cancer risk [35]. Our study uses a larger samples size to support the findings of Rosenthal and also clarifies the interaction between *XRCC1* and *TP53* in influencing cervical cancer risk in China. It is likely that the inconsistency in population molecular genetics studies on the association between *TP53* and cervical carcinoma is due in part to differences in the status and intensity of HPV infection and mutation rates of this virus in diverse regions. HPV infection and *TP53* mutation are not independent high-risk factors for cervical cancer, but *TP53* does promote CIN progression to cervical cancer. The interaction of genetic, intrinsic, and environmental factors is causally linked to the occurrence and development of cervical cancer.

Our results highlight the role of heterozygote rs25487 and rs1042522 variants in the susceptibility to cervical cancer within the Chinese southwestern population. It must be noted, however, that no obvious interaction between the minor alleles was detected when analyzing the cervical cancer group. We speculate that this phenomenon may result from the high lethality rate associated with the minor allele that may prevent significant results from being derived from data analysis of small sample pools. Of our samples, 31.71% (negative control group: 9.76, elderly patients: 7.32, cases group: 14.63) simultaneously exhibit an “A” allele of *XRCC1* and a “C” allele of *TP53*. There were only 7/328 (2.13%) subjects exhibiting minor frequency in both the *XRCC1* and *TP53* genes. Additionally, differences in HPV infection history within our experimental group may have influenced our results. Cervical cancer risk is associated with high-risk HPV infection, and the penetrance of genetic high-risk factors is based on HPV infection [36]. Given this, identical status or intensity of HPV exposure within a given group is a prerequisite for statistical analysis. In clinical practice, it is difficult to meet this prerequisite as HPV infection rates and subtype distributions in various regions are different, with the exception of the cancer group [37, 38]. According to our clinical sample data, the HPV positive infection rate of our groups is 58%. Based on the observed high HPV infection rates in Chongqing, we propose that mortality rates due to cervical cancer will increase when *XRCC1* and *TP53* are simultaneously mutated [37]. Therefore, the influence of the SNP-SNP interaction between *XRCC1* and *TP53* in the context of cervical cancer is meaningful only when patients exhibit heterozygosity at the *XRCC1* and *TP53* loci (Tables 3 and 4).

Genetic testing combined with primary high-risk HPV testing has the potential to reduce the morbidity and mortality associated with cervical cancer. Based on our data, clinicians will gain critical insight into the mechanisms underlying the development of cervical cancer to provide novel approaches for the treatment of this disease.

## Conclusions

Our novel approach of using an elderly control group (> 80 yrs) with no history of cervical cancer allowed us to confirm a significant association between two SNPs, *XRCC1* rs25487 and *TP53* rs1042522, and the development of cervical cancer within the southwestern Chinese population. Additionally, we demonstrate the novel finding that no mutation of *FGFR3* exists in the southwestern Chinese population. Finally, we clarify the inconsistent conclusions regarding the relationship between *TP53* and cervical cancer by using a population molecular genetics approach.

## Additional files

Additional file 1: The inclusion and exclusion criteria of subjects. (DOCX 15 kb)

Additional file 2: Patient demographics and clinical characteristics of patients studied. (DOCX 15 kb)

Additional file 3: MAMA-PCR and Sanger sequencing for gene typing. (BMP 8580 kb)

## Abbreviations

BER: Base excision repair
CI: Confidence interval
FGFR3: Fibroblast growth factor receptor 3
GMDR: Generalized multifactor dimensionality reduction
HPV: Human papillomavirus
HWE: Hardy-Weinberg equilibrium
MAMA-PC: Mutation analysis of mismatch amplification
OR: Odds ratio
SD: Standard deviation
SNP-SNP: Single nucleotide polymorphisms-single nucleotide polymorphisms
STD: Sexually transmitted disease
TP53: Tumor protein p53
XRCC1: X-ray repair cross complementing 1
χ^2^: Chi-square
