# Supplementary material for: Interaction between TP53 and XRCC1 increases susceptibility to cervical cancer development: a case control study
Source: BMC Cancer. 2019 Jan 7;19:24. doi: 10.1186/s12885-018-5149-0 (PMC6323714; doi:10.1186/s12885-018-5149-0)
Supplement: Supplementary file 1 — The inclusion and exclusion criteria of subjects. (DOCX 15 kb) [file 12885_2018_5149_MOESM1_ESM.docx]

The inclusion and exclusion criteria of subjects

Inclusion Criteria

- The subjects had similar ethnic and geographical background.
- The ages of the enrolled subjects in cases and the negative controls groups were in the range of 20~60 years old.
- Histologically, the patients were all confirmed as primary cervical cancer and recruited in case group.
- The elderly patients above the age of 80 and proven none cervical cancer history all their lives were enrolled in elder control group.

Exclusion Criteria

- The subjects should be no bone marrow transplant.
- Presence of cardiovascular pathologies or other diseases
- The negative controls should none positive findings during the gynecological examination, no history of cancer, age matching to the patients and residence in Chongqing.
