# Supplementary material for: Interaction between TP53 and XRCC1 increases susceptibility to cervical cancer development: a case control study
Source: BMC Cancer. 2019 Jan 7;19:24. doi: 10.1186/s12885-018-5149-0 (PMC6323714; doi:10.1186/s12885-018-5149-0)
Supplement: Supplementary file 2 — Patient demographics and clinical characteristics of patients studied. (DOCX 15 kb) [file 12885_2018_5149_MOESM2_ESM.docx]

Patient demographics and clinical characteristics of patients studied.

| HPV Status | No. of Patients | Age | Associated Diseases | Previous Screening | Previous STD |
| --- | --- | --- | --- | --- | --- |
| HPV positive | 140 | 60.68±1.81 | YES:66 NO:74 | YES:8 NO:132 | YES:11 NO:129 |
| HPV negative | 197 | 41.58±0.94 | YES:52 NO:145 | YES:14 NO:183 | YES:8 NO:189 |
| P |  | < 0.0001 | < 0.0001 | 0.61 | 0.14 |

*3 enrolled subjects were excluded from the analysis due to the missed clinical information for the HPV infection history.
