# Supplementary figures and images for: Interaction between TP53 and XRCC1 increases susceptibility to cervical cancer development: a case control study
Source: BMC Cancer. 2019 Jan 7;19:24. doi: 10.1186/s12885-018-5149-0 (PMC6323714; doi:10.1186/s12885-018-5149-0)

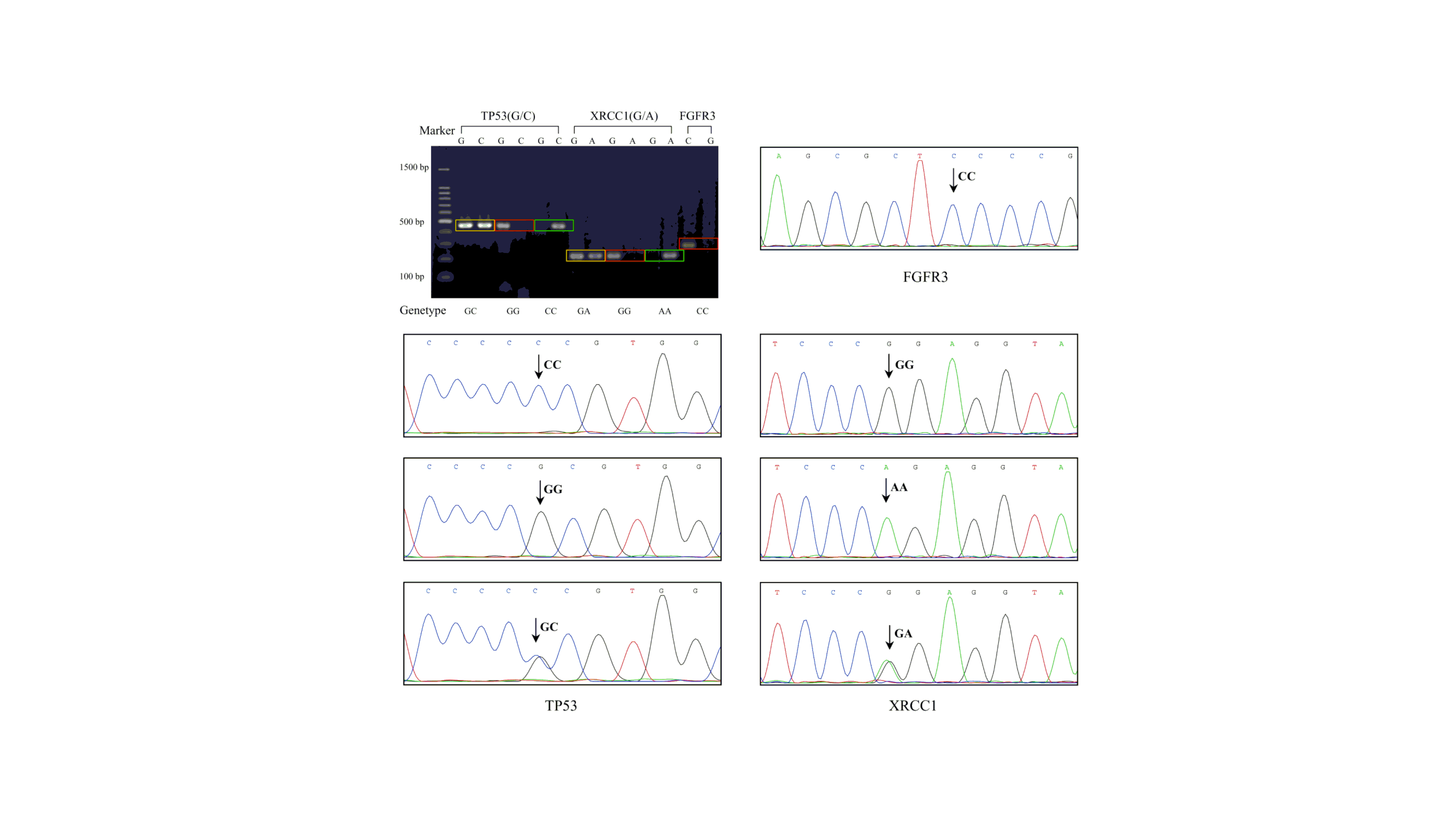

Supplement: Supplementary file 3 — MAMA-PCR and Sanger sequencing for gene typing. (BMP 8580 kb) [file 12885_2018_5149_MOESM3_ESM.bmp]
